# Comparison of Intramedullary Nail and Volar Locking Plate for Distal Radius Fractures: A Systematic Review and Meta-Analysis of Randomised Controlled Trials

**DOI:** 10.7759/cureus.17972

**Published:** 2021-09-14

**Authors:** Zehong Chen, Yinan Zhu, Wei Zhang, Hassan Eltagy, Sherif Elerian

**Affiliations:** 1 Trauma and Orthopaedics, Sandwell General Hospital, Birmingham, GBR; 2 Oral and Maxillofacial Surgery, University College Hospital, London, GBR; 3 Orthopaedic Surgery, Tan Tock Seng Hospital, Singapore, SGP

**Keywords:** distal radius fracture, distal end radius plating, intramedullary nail, volar locking plate, meta-analysis

## Abstract

Operative intervention with a volar locking plate (VLP) is currently the gold standard for the fixation of distal radius fractures. Intramedullary nailing (IMN) of the distal radius is a novel technique that aims to reduce soft tissue complications due to a smaller surgical incision while maintaining the benefits of a rigid fracture fixation. The aim of this systematic review and meta-analysis was to investigate the functional, clinical, and radiological outcomes of all published randomised controlled trials (RCTs) comparing patient outcomes of VLP and IMN in distal radius fracture fixation. Three databases (Ovid MEDLINE, EMBASE, and Cochrane Library) were searched in July 2021. The inclusion criteria were RCTs comparing fixation of extra-articular or simple intra-articular distal radius with VLP or IMN and availability of full text in English. Children under the age of 18 were excluded. Seven trials with a total of 398 patients were included in this meta-analysis. The meta-analysis showed that there were improved short-term clinical outcomes favouring IMN, although there were no significant differences in terms of functional, radiological, and long-term clinical outcomes. Analysis showed that outcomes of IMN are comparable with VLP for fixation of extra-articular and simple intra-articular distal radius fractures. However, these results should be interpreted with caution due to the small sample size. We recommend that further high-quality trials are required to establish the role of IMN in distal radius fixation.

## Introduction and background

Distal radius fractures are one of the most common fractures and they account for approximately 18% of all fractures [1]. Distal radius fractures can be managed conservatively or surgically, and the management option is dependent on the patient’s functional needs, co-morbidities, and fracture pattern. The American Academy of Orthopaedic Surgeons recommends that operative treatment should be undertaken for non-geriatric patients with radial shortening of more than three millimetres, a dorsal tilt of more than ten millimetres, or intra-articular step of more than two millimetres [2,3].

Operative intervention using a volar locking plate (VLP) is currently the gold standard approach for distal radius fractures because it is able to restore intra-articular alignment and volar tilt by direct visualisation of the fracture whilst providing a stable and rigid fixation that reduces the rate of post-traumatic osteoarthritis [4,5]. Despite the benefits of VLP, it is associated with complications including tenosynovitis, tendon irritation, rupture requiring hardware removal, and complex regional pain syndrome [6]. Due to the larger incision site, there is also greater surgical exposure and soft tissue irritation, which may cause infection and chronic pain [7].

The use of intramedullary nail (IMN) in distal radius fractures is a novel technique that aims to reduce soft tissue complications due to a smaller surgical incision whilst maintaining the benefits of a rigid fracture fixation. It is indicated in displaced extra-articular or simple intra-articular distal radius fractures but should not be used when articular fragments are small and the fracture is not reducible by closed or percutaneous measures. The purported benefits of IMN include reduced soft-tissue disruption, hence allowing improved bone and wound healing and reduced risk of complications such as carpal tunnel syndrome (CTS), hardware prominence, and tendon irritation [8]. Despite all the hypothesised advantages of IMN and its routine use in fractures of the femur, tibia, humerus, and paediatric forearm fractures, it is not commonly performed for distal radius fractures in adults.

Although there were two meta-analyses published on this topic, one of them included retrospective trials which could have introduced a degree of bias [9,10]. There have also been more trials published since. In our systematic review and meta-analysis, we aimed to investigate the functional, clinical, and radiological outcomes of all published randomised controlled trials (RCTs) comparing VLP and IMN in distal radius fracture fixation.

## Review

Methods

Literature Search Strategy

Three databases (Ovid MEDLINE, EMBASE, and Cochrane Library) were searched in July 2021. The search was performed according to the Preferred Reporting Items for Systematic Reviews and Meta-Analysis (PRISMA) guidance. The search strategy can be found in the Appendices.

Inclusion and Exclusion Criteria

The inclusion criteria were RCTs comparing fixation of extra-articular or simple intra-articular distal radius with VLP or IMN and availability of full text in English. Children under the age of 18 were excluded.

Study Selection

Two authors independently applied the search strategy and screened for all titles and abstracts. The bibliographies were also screened for any potentially relevant studies. The full paper was reviewed when there were any uncertainties regarding study eligibility. Any disagreements were discussed and resolved by consensus.

Data Extraction

A data extraction form was designed according to the Cochrane Collaboration’s data collection form for intervention review [11]. Two authors performed data extraction independently. Any discrepancies in data collection were discussed and resolved by consensus.

Risk of Bias

The Cochrane Collaboration’s risk of bias tool was used to evaluate the risk of bias in every individual study. The criteria that were assessed for bias were: random sequence generation, allocation concealment, blinding of participants and personnel, blinding of outcome assessment, incomplete outcome data, selective reporting, and other sources of biases. The biases were reported as low risk, high risk, or unclear risk. Two authors analysed the papers independently and any discrepancies were discussed and resolved by consensus. 

Data Analysis

Results were analysed using Revman 5.4 (The Cochrane Collaboration, London, UK). All continuous data were presented using mean and standard deviation (SD). Continuous outcomes were analysed with pooled inverse variance weighting and presented as standard mean difference (SMD) or mean difference (MD) with a 95% confidence interval (CI). All analysis was performed with fixed effects models. Heterogeneity was considered significant if I2 >50%. A p-value <0.05 was considered statistically significant.

Outcome Measures

The primary outcome investigates functional outcomes of the upper extremity after IMN versus VLP in distal radius fractures. This was recorded using Patient Reported Outcome Measures such as the Disabilities of the Arm, Shoulder and Hand (DASH) score or Gartland and Werley Score (GWS). Secondary outcome measures were short-term (six weeks) and long-term (one to two years) clinical outcomes (range of movement [ROM]) and radiological outcomes (volar tilt, ulnar variance, radial height, and radial inclination). 

Results

Study Selection

**Figure 1:**
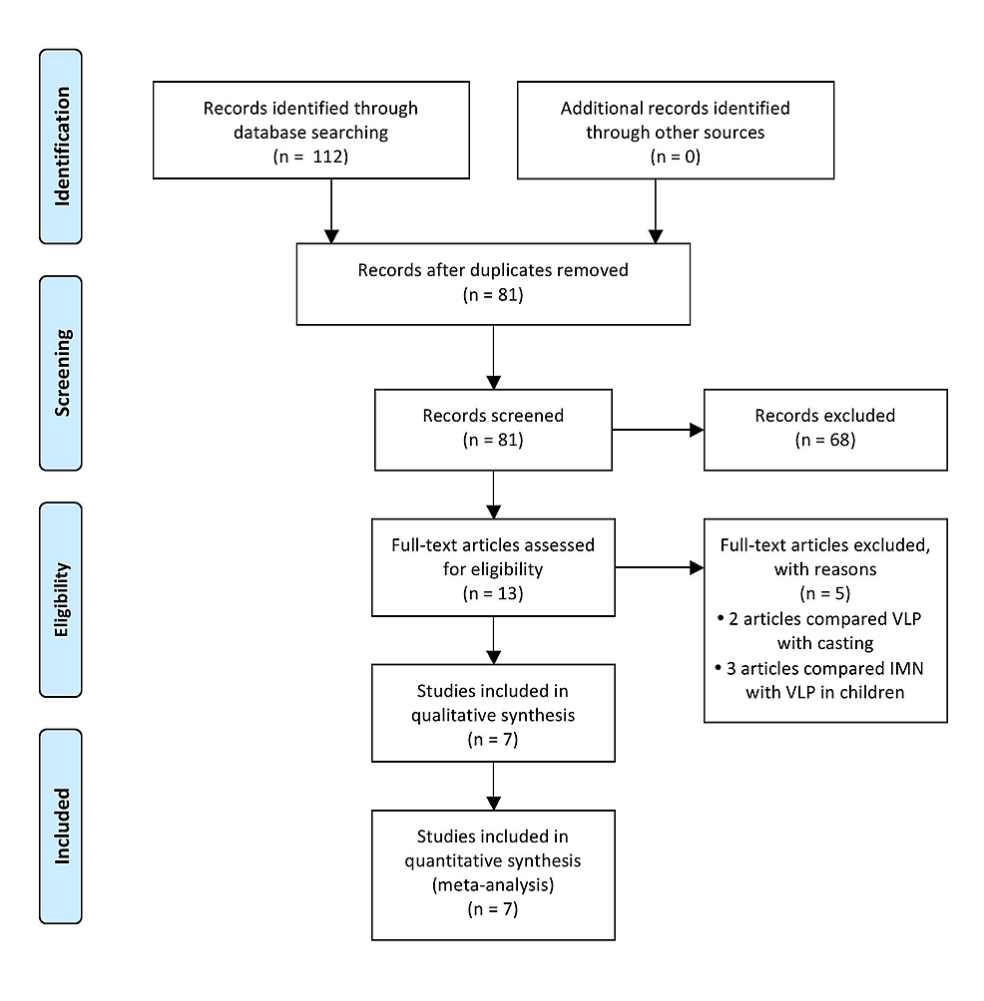
PRISMA flow diagram PRISMA: Preferred Reporting Items for Systematic Reviews and Meta-Analysis

The literature search identified 99 articles altogether, and one article was identified from the bibliography of the papers reviewed. There were 81 articles after deduplication and 10 trials were shortlisted after browsing through their titles and abstracts (Figure 1).

Seven trials with a total of 398 patients were included in this meta-analysis as they met the inclusion criteria (Table 1).

**Table 1:** Characteristics of included randomised controlled trials DASH: Disabilities of the arm, shoulder and hand; GWS: Gartland-Werley score; MHQ: Michigan hand outcomes questionnaire; QuickDASH: Quick disabilities of the arm, shoulder and hand; ROM: Range of movement; VAS: Visual analogue score; RCT: Randomised controlled trials

Authors	Year	Country	n	Type of study	Male (%)	Mean age (years)	Type of fracture	Outcomes	Duration of follow-up
Aita et al. [12]	2014	Brazil	48	RCT	45.8	36	Extra-articular distal radius fracture	Radiographic, ROM, grip strength, DASH score, VAS, complications	12 months
Chappuis et al. [13]	2011	Belgium	31	RCT	-	72	Extra-articular distal radius fracture with dorsal tilt	Radiographic, ROM, tightening strength, DASH score, mayo clinic score, complications	6 months
Gradl et al. [14]	2014	Germany	121	RCT	9	62	Extra-articular distal radius fracture with dorsal tilt > 20°	Radiographic, ROM, grip strength, VAS, castaing score, GWS	24 months
Gradl et al. [15]	2016	Germany	25	RCT	14.3	64	Intra-articular distal radius fracture AO type C2.1	Radiographic, ROM, grip strength, VAS, castaing score, GWS	24 months
Plate et al. [16]	2015	USA	45	RCT	26.7	55	Extra-articular distal radius fracture AO type A	Radiographic, ROM, QuickDASH score, MHQ, pain medication	24 months
Safi et al. [17]	2013	Czech Republic	61	RCT	21.3	57	Unstable extra-articular distal radius fracture AO type A1, A2 and unstable simple intra-articular fracture B1.1, B1.2	Radiographic, ROM, DASH score, mayo wrist score	12 months
Zehir et al. [18]	2014	Germany	64	RCT	18.8	47	Unstable extra-articular distal radius fracture AO type A2.2, 2.3, 3.1 and simple intra-articular fracture C2.1, 2.2	Radiographic, ROM, stewart score, GWS	12-13 months

Risk of Bias Analysis

**Figure 2:**
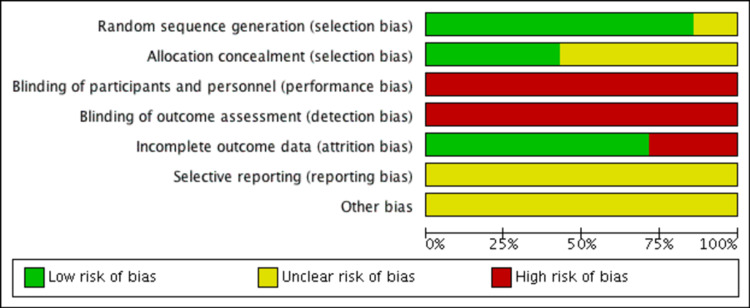
Risk of bias graph

**Figure 3:**
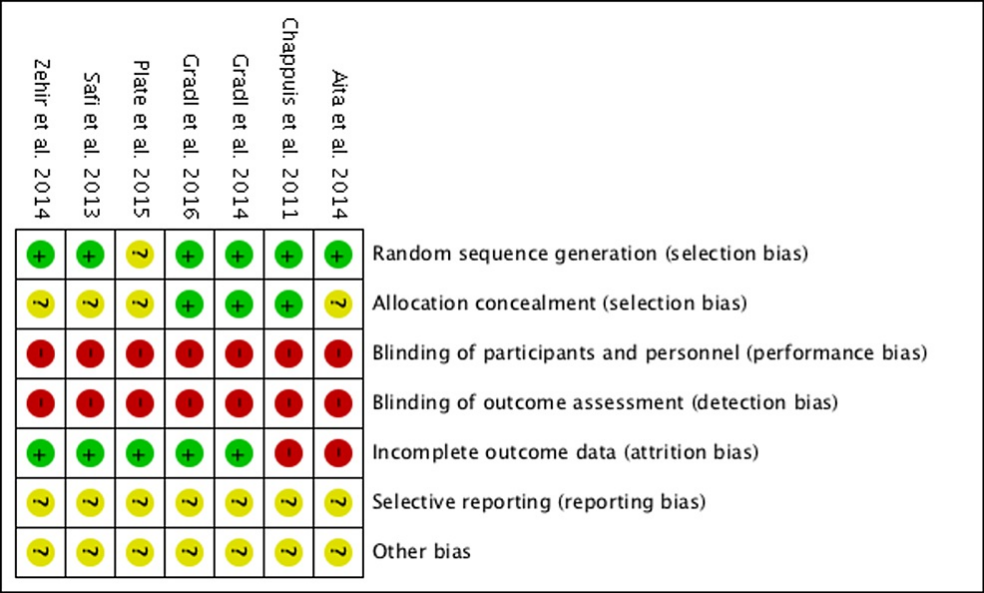
Risk of bias summary Aita et al. [12], Chappuis et al. [13], Gradl et al. [14], Gradl et al. [15], Plate et al. [16], Safi et al. [17], Zehir et al. [18]

All studies were described as low risk, high risk, or unclear risk in each domain (Figures 2, 3). Six RCTs described their process of randomisation, while one did not elaborate on the process of randomisation. Three RCTs concealed patient allocation prior to assignment by using sealed opaque envelopes. All studies had a high risk of bias with no blinding of participants and personnel due to the intervention undertaken, as both patients and surgeons needed to know the operation involved to consent and perform it. Blinding of outcome assessment was not possible as well, as surgical scars and radiographic imaging would inform the assessor of the intervention performed, and hence was deemed high risk of bias in all studies. Two RCTs had incomplete outcome data; one did not provide data for one of the outcomes stated in the methodology, while the other did not clarify if the data given were mean or median scores, and there were no standard deviation data provided which did not allow the data to be used in the analysis [12]. There was an unclear risk for all studies for selective reporting, as none of the studies reported publishing a protocol in advance.

Functional Outcome

**Figure 4:**
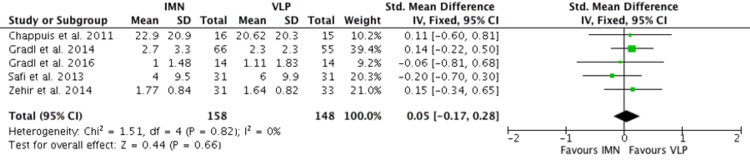
Functional outcome Chappuis et al. [13], Gradl et al. [14], Gradl et al. [15], Safi et al. [17], Zehir et al. [18] IMN:  Intramedullary nail; VLP: Volar locking plate

Five RCTs [13-18] measured functional outcomes with either the DASH score or the GWS score following either intervention. There were a total of 316 patients, and the SMD was used to calculate the forest plot (Figure 4). This did not show any statistical difference in the functional outcome of IMN compared to VLP (p=0.66).

Short-Term Clinical Outcome

**Figure 5:**
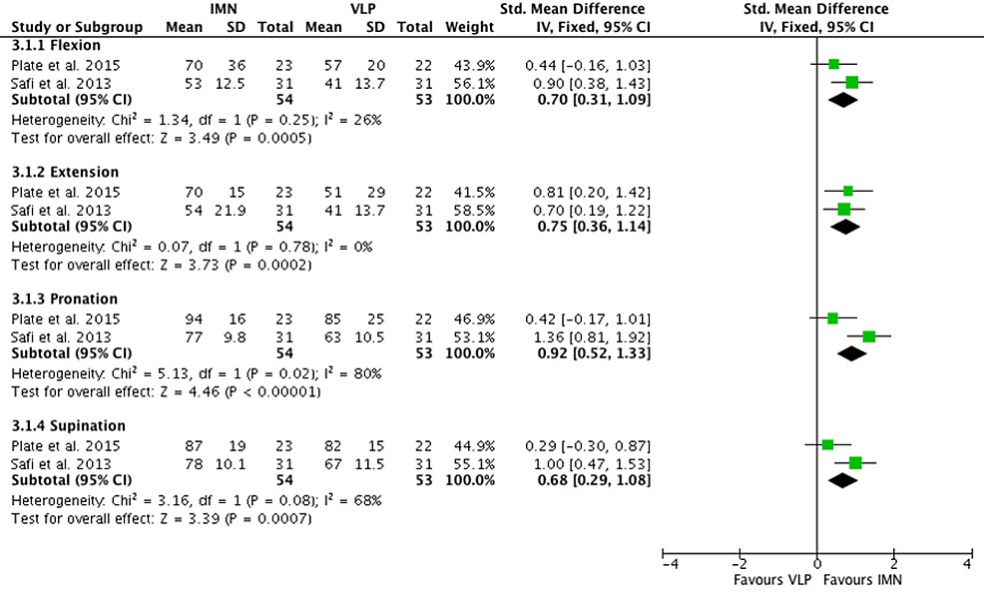
Short-term clinical outcome Plate et al. [16], Safi et al. [17] IMN:  Intramedullary nail; VLP: Volar locking plate

Two RCTs [15,16] investigated short-term clinical outcomes measuring ROM of flexion, extension, pronation, and supination six weeks after the operation. Plate et al. measured this in % of the contralateral side, while Safi et al. measured this in degrees, and the SMD was used to calculate this outcome (Figure 5). There was greater ROM in all planes of wrist movements at six weeks after intervention in patients who underwent IMN as compared to VLP (flexion: mean 0.70 95% CI 0.31-1.09, extension: mean 0.75 95% CI 0.36-1.14, pronation: mean 0.92 95% CI 0.52-1.33, supination: mean 0.68 95% CI 0.29-1.08). However, the analysis did show high heterogeneity in pronation and supination (I2=80% and 68% respectively).

Long-Term Clinical Outcome

**Figure 6:**
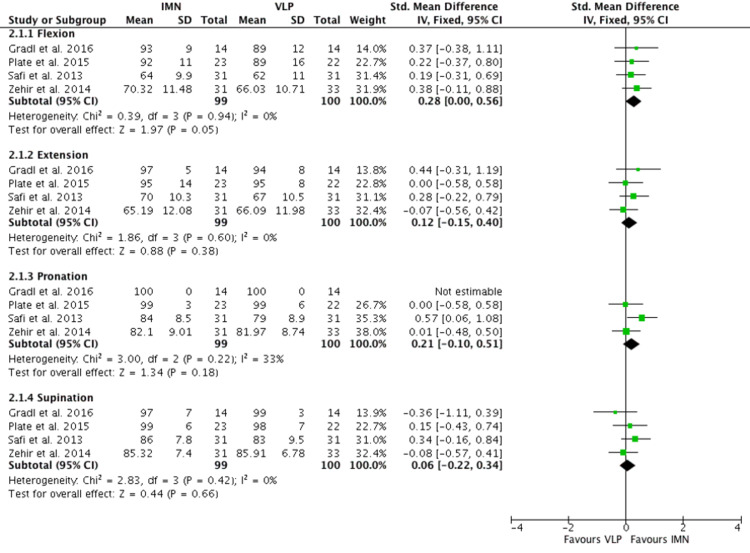
Long-term clinical outcome Gradl et al. [15], Plate et al. [16], Safi et al. [17], Zehir et al. [18] IMN:  Intramedullary nail; VLP: Volar locking plate

Four RCTs [15-18] investigated clinical outcomes measuring ROM of flexion, extension, pronation, and supination in a total of 99 patients’ wrists after the operation. These were measured one to two years after the operation. Only results from four papers could be used, as data from the other RCTs were incomplete (Figure 6). Gradl et al. and Plate et al. [15, 16] measured ROM based on % of the contralateral side, while Safi et al. and Zehir et al. [17, 18] measured this ROM in degrees, as such SMD was used for the forest plot. There were no statistical differences in flexion, extension, pronation and supination between IMN and VLP (flexion: mean 0.28 95% CI 0.00 - 0.56, extension: mean 0.12 95% CI -0.15-0.40, pronation: mean 0.21 95% CI -0.10-0.51, supination: mean 0.06 95% CI -0.22-0.34).

Radiological Outcome

**Figure 7:**
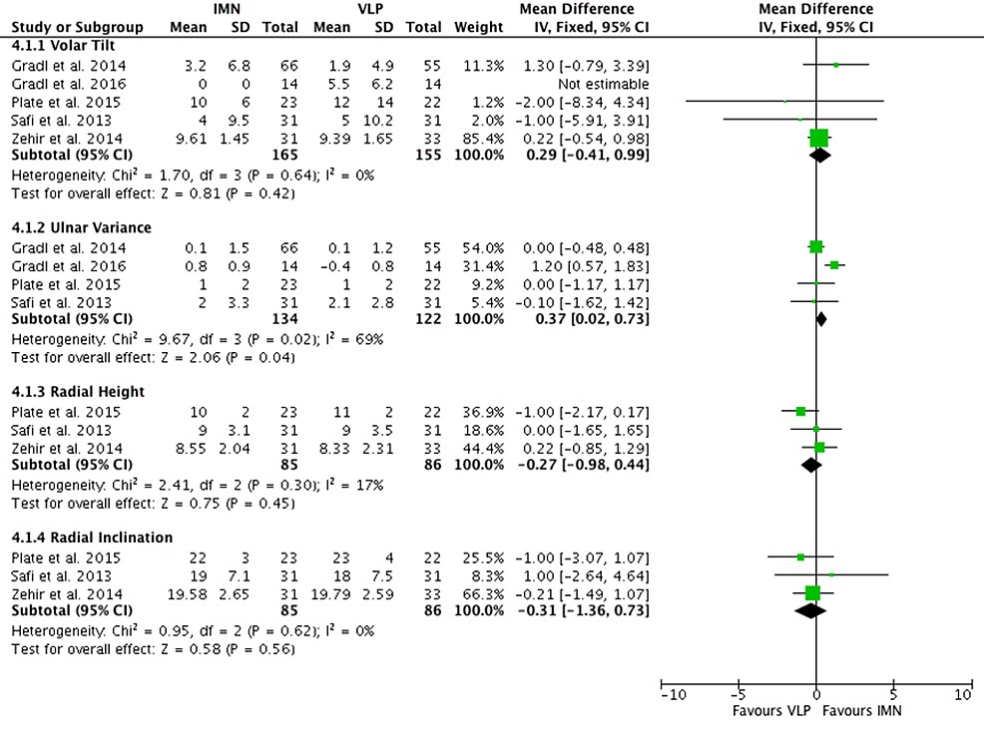
Radiological outcome Gradl et al. [14], Gradl et al. [15], Plate et al. [16], Safi et al. [17], Zehir et al. [18] IMN:  Intramedullary nail; VLP: Volar locking plate

Three to five RCTs [13-17] measured radiological outcomes of 85 patients after having the intervention. The radiological outcomes being investigated were volar tilt, ulnar variance, radial height, and radial inclination (Figure 7). There were no statistical differences between IMN or VLP in volar tilt, radial height, or radial inclination. Only ulnar variance favoured IMN over VLP (mean 0.37 95% CI 0.02-0.73), however, heterogeneity was high (I2 = 69%).

Discussion

The findings from our meta-analysis showed that IMN and VLP were comparable in radiological, clinical, and functional outcomes, although those who received IMN had better short-term clinical outcomes. 

The improved short-term clinical outcome of IMN may be due to its mechanics. IMN functions as a load-sharing device and is a stronger construct compared to VLP [19,20]. This allows early wrist movement which reduces stiffness in the hand and improves ROM in the short term. However, in the long term, there appears to be no significant difference, signifying that this difference eventually evens out with appropriate hand therapy, exercises, and encouragement of patients to resume activities of daily living when appropriate. This highlights the importance of hand therapy and rehabilitation to improve the patient’s range of movement and outcomes following operation [21]. It is crucial to note that in all the RCTs that were included in this study, only extra-articular or simple intra-articular distal radius fractures were fixed with IMN. This is because it is important to restore the articular surface of the wrist joint to reduce rates of post-traumatic osteoarthritis, and anatomical reduction is not possible with an IMN [22].

There were two other meta-analyses comparing IMN with VLP in distal radius fractures, by Zhang et al. and Wang et al. [9,10]. There have since been more trials, and these were included in our paper. Similar to our findings, Zhang et al. found that there were no significant differences between either fixation method in terms of functional score, range of movement, and radiographic parameters. However, they demonstrated that IMN performed better than VLP in functional scoring in the post-operative period from six weeks to three months [10]. On closer scrutiny, Zhang et al. came to this conclusion only with the results from one RCT reported by Safi et al. [9,17]. Wang et al. observed similar findings to our meta-analysis, with no significant differences in the functional score, radiographic parameters, and range of movement, although he showed that there were higher incidents of CTS in VLP [10]. Hence, our main findings were consistent with others stated in the literature. Furthermore, it is also crucial to investigate the rate of complications to decide on the superior fixation method. 

Complications arising from procedures can result in additional time, patient dissatisfaction, and an increase of up to five times the initial cost of a similar operation without any complications [23]. The common complications arising from a fixation of the distal radius include tendon injury, CTS, radial nerve paraesthesia, chronic regional pain syndrome, and metalwork irritation. Jordan et al. reported that due to the different approaches between both fixations, the complications were different, and it was more common for IMN to result in neuropraxia of the superficial radial nerve, tenosynovitis, and irritation from metalwork. He described that the complication rates of IMN was higher than expected, although it was not of statistical significance [24]. Zhang et al. showed that there were no significant differences observed for any post-operative complication between IMN or VLP apart from rates of CTS that was more likely to occur in patients who had VLP (8.7%) compared to IMN (0.8%) [9]. Wang et al. corroborated this and showed that there was an increased risk of CTS in VLP (6%-12%) [10]. Interestingly, a retrospective study by Lee et al. of 1955 cases had published that rates of CTS were not as high, at 1.84% [25]. This might be due to the low number of patients included in the aforementioned studies that could have skewed the results and stronger statistical power would be needed to accurately objectively comment on the rates of complication of VLP. With this in mind, perhaps IMN should be preferred over VLP due to similar outcomes and lower complications of CTS.

Limitations

There were limited RCTs with a modest total of 398 patients despite searching through all literature available, and this may be due to the novelty of using IMN for distal radius fractures. Additionally, one of the trials failed to publish their findings for standard deviation of all the data and therefore could not be included in our study. There was also high heterogeneity in a few of the outcomes that may affect interpretation of results.

## Conclusions

The results from this paper show that the outcomes of IMN are comparable with VLP for the fixation of extra-articular and simple intra-articular distal radius fractures. There were no significant differences in terms of functional, radiological, and long-term clinical outcomes. These results should be interpreted with caution due to the small sample size. We recommend that further high-quality trials are required to establish the role of IMN in distal radius fixation.

## Appendices

Search strategies for MEDLINE, EMBASE and Cochrane Library (Tables 2-4).

**Table 2:** Search strategy in MEDLINE MeSH: Medical Subject Headings

1	Radius fracture.mp or Radius Fractures [MeSH]	10448
2	Fracture Fixation, Intramedullary [MeSH] or Bone Nails [MeSH] or Nails.mp or Nails [MeSH] or Internal Fracture Fixation.mp or Fracture Fixation, Internal [MeSH]	66682
3	Volar Locking.mp or Bone Plate [MeSH]	19127
4	#1 and #2 and #3	1424
5	Limit #4 to (English language and “all adults (19 plus years)” and randomized controlled trial)	80

**Table 3:** Search strategy in EMBASE MeSH: Medical Subject Headings

1	Radius fracture.mp or Radius Fractures [MeSH]	10686
2	Intramedullary fracture fixation.mp or intramedullary nailing [MeSH] or bone nail.mp or bone nail [MeSH]	17831
3	Volar Locking.mp or Bone Plate [MeSH]	17142
4	#1 and #2 and #3	122
5	limit #4 to (English language and randomized controlled trial and (adult <18 to 64> or aged <65+ years>))	11

**Table 4:** Search strategy in Cochrane MeSH: Medical Subject Headings

1	MeSH descriptor: [Radius Fractures] explode all trees	609
2	MeSH descriptor: [Fracture Fiation, Intramedullary] explode all trees	358
3	MeSH descriptor: [Bone Plates] explode all trees	616
4	#1 and #2 and #3	8

## References

[1] Baron JA, Karagas M, Barrett J, Kniffin W, Malenka D, Mayor M, Keller RB (1996). Basic epidemiology of fractures of the upper and lower limb among Americans over 65 years of age. Epidemiology.

[2] (2021). Management of distal radius fractures. https://www.aaos.org/globalassets/quality-and-practice-resources/distal-radius/drfcpg.pdf.

[3] Lichtman DM, Bindra RR, Boyer MI (2010). Treatment of distal radius fractures. J Am Acad Orthop Surg.

[4] Seigerman D, Lutsky K, Fletcher D (2019). Complications in the management of distal radius fractures: how do we avoid them?. Curr Rev Musculoskelet Med.

[5] Karantana A, Downing ND, Forward DP (2013). Surgical treatment of distal radial fractures with a volar locking plate versus conventional percutaneous methods: a randomized controlled trial. J Bone Joint Surg Am.

[6] Lattmann T, Meier C, Dietrich M, Forberger J, Platz A (2011). Results of volar locking plate osteosynthesis for distal radial fractures. J Trauma.

[7] Bentohami A, de Burlet K, de Korte N, van den Bekerom MP, Goslings JC, Schep NW (2014). Complications following volar locking plate fixation for distal radial fractures: a systematic review. J Hand Surg Eur Vol.

[8] Tan V, Capo J, Warburton M (2005). Distal radius fracture fixation with an intramedullary nail. Tech Hand Up Extrem Surg.

[9] Zhang B, Chang H, Yu K (2017). Intramedullary nail versus volar locking plate fixation for the treatment of extra-articular or simple intra-articular distal radius fractures: systematic review and meta-analysis. Int Orthop.

[10] Wang J, Zhang L, Ma J, Yang Y, Jia H, Ma X (2016). Is intramedullary nailing better than the use of volar locking plates for fractures of the distal radius? A meta-analysis of randomized controlled trials. J Hand Surg Eur Vol.

[11] (2021). Cochrane Handbook for Systematic Reviews of Interventions Version 6.2. https://training.cochrane.org/handbook/current.

[12] Aita MA, Vieira Ferreira CH, Schneider Ibanez D (2014). Randomized clinical trial on percutaneous minimally invasive osteosynthesis of fractures of the distal extremity of the radius. Rev Bras Ortop.

[13] Chappuis J, Bouté P, Putz P (2011). Dorsally displaced extra-articular distal radius fractures fixation: dorsal IM nailing versus volar plating. A randomized controlled trial. Orthop Traumatol Surg Res.

[14] Gradl G, Mielsch N, Wendt M, Falk S, Mittlmeier T, Gierer P, Gradl G (2014). Intramedullary nail versus volar plate fixation of extra-articular distal radius fractures. Two year results of a prospective randomized trial. Injury.

[15] Gradl G, Falk S, Mittlmeier T, Wendt M, Mielsch N, Gradl G (2016). Fixation of intra-articular fractures of the distal radius using intramedullary nailing: a randomized trial versus palmar locking plates. Injury.

[16] Plate JF, Gaffney DL, Emory CL (2015). Randomized comparison of volar locking plates and intramedullary nails for unstable distal radius fractures. J Hand Surg Am.

[17] Safi A, Hart R, Těknědžjan B, Kozák T (2013). Treatment of extra-articular and simple articular distal radial fractures with intramedullary nail versus volar locking plate. J Hand Surg Eur Vol.

[18] Zehir S, Calbiyik M, Zehir R, Ipek D (2014). Intramedullary repair device against volar plating in the reconstruction of extra-articular and simple articular distal radius fractures; a randomized pilot study. Int Orthop.

[19] Bong MR, Kummer FJ, Koval KJ, Egol KA (2007). Intramedullary nailing of the lower extremity: biomechanics and biology. J Am Acad Orthop Surg.

[20] Brooks KR, Capo JT, Warburton M, Tan V (2006). Internal fixation of distal radius fractures with novel intramedullary implants. Clin Orthop Relat Res.

[21] Ikpeze TC, Smith HC, Lee DJ, Elfar JC (2016). Distal radius fracture outcomes and rehabilitation. Geriatr Orthop Surg Rehabil.

[22] Fitoussi F, Ip WY, Chow SP (1997). Treatment of displaced intra-articular fractures of the distal end of the radius with plates. J Bone Joint Surg Am.

[23] Vonlanthen R, Slankamenac K, Breitenstein S (2011). The impact of complications on costs of major surgical procedures: a cost analysis of 1200 patients. Ann Surg.

[24] Jordan RW, Saithna A (2015). Defining the role of intramedullary nailing for fractures of the distal radius: a systematic review. Bone Joint J.

[25] Lee JH, Lee JK, Park JS (2020). Complications associated with volar locking plate fixation for distal radius fractures in 1955 cases: a multicentre retrospective study. Int Orthop.

